# Biosolids in Washington State: Putting per‐ and poly‐fluorinated alkyl substances concerns into a real‐world context

**DOI:** 10.1002/jeq2.70279

**Published:** 2026-10-18

**Authors:** Sally Brown, Scott Mansell

**Affiliations:** ^1^ School of Environmental and Forest Sciences University of Washington Seattle Washington USA; ^2^ Research and Innovation Division Clean Water Services Portland Oregon USA

## Abstract

Washington State has a history of beneficial use of municipal biosolids. The Department of Ecology recently conducted a voluntary survey of per‐ and poly‐fluorinated alkyl substances (PFAS) in biosolids. Results of that survey are presented and compared with the results from mandatory sampling in Michigan. For the majority of the utilities that participated, residential flow was found to be the primary source of PFAS for biosolids suggesting that industrial source control will not limit their presence in biosolids. Distribution of PFAS was similar to that identified in the Michigan sampling. The results of the sampling were put into the context of current biosolids end use to evaluate the potential for increased human exposure to PFAS as a consequence of beneficial use of biosolids. This was done in the context of identifying the potential for a highly exposed individual (HEI) as is traditionally done in risk assessment and was recently done in a draft Environmental Protection Agency risk assessment. The majority of end use sites; dryland wheat and commercial tree plantations, show no realistic potential for a HEI. Others, such as garden vegetables and animal feed crops, have identified exposure pathways and may merit additional sampling.

## Core Ideas

- Per‐ and poly‐fluorinated alkyl substances (PFAS) concentrations in 44 biosolids in Washington State were generally similar to those reported at water recovery facilities in Michigan, suggesting that there will be measurable concentrations of PFAS in a majority of biosolids.
- The total oxidizable precursor assay did not reliably predict individual PFAS concentrations as measured by the Environmental Protection Agency 1633 protocol.
- End use of biosolids in Washington State suggests minimal risk for identification of a highly exposed individual for the majority of beneficial uses.

## Plain Language Summary

The presence of poly‐fluorinated alkyl substances (PFAS) in municipal biosolids has resulted in significant concerns re the safety of beneficial use. Widespread use of these substances in common household products suggests that they will be detected in biosolids. This study reports on a voluntary sampling of biosolids in Washington State. Washington has encouraged beneficial use of biosolids. A majority of the biosolids produced in the state are applied to soils. PFAS was detected in all biosolids sampled with a third of the facilities having concentrations suggesting potential industrial sources. We looked at how biosolids are used in the state to see if their use might result in increased exposure. Most of the biosolids are applied to dryland wheat and commercial forestry and have no realistic exposure pathways. Other uses such as urban agriculture and animal feed may merit additional sampling. These results show the importance of understanding PFAS in biosolids in a real‐world context to assess potential risk.

**Abbreviations**
PFAS: per‐ and poly‐fluorinated alkyl substances
TOP: total oxidizable precursors

## 1 INTRODUCTION

Wastewater treatment and its associated products (effluent, recycled water, and municipal biosolids) have received significant attention as sources of perfluorinated compounds to the environment (Ghaznavi et al., 2023; Washington et al., 2010). Initial focus was on biosolids with elevated per‐ and poly‐fluorinated alkyl substances (PFAS) due to industrial discharges into the system. For example, elevated PFAS in biosolids were first noted in Decatur, AL, the site of a 3 M manufacturing plant (Washington et al., 2010). However, PFAS are present in biosolids even without any industrial sources. A more recent study measured concentrations of PFAS in treatment works and identified domestic flow as the primary source (Lin et al., 2024). While concentrations here were significantly lower than in industrially or historically impacted biosolids, mean concentrations were above the levels used in the recent United States Environmental Protection Agency (US EPA) draft risk assessment (US EPA, 2025). This suggests that influent from homes will result in measurable PFAS concentrations in municipal biosolids.

The first survey of PFAS concentrations in biosolids was conducted on composite samples collected in 2001 prior to the phase out of perfluorooctanoic acid (PFOA) and perfluorooctane sulfonic acid (PFOS) (Venkatesan & Halden, 2013). The authors found PFOS concentrations of 403 ± 127 ng g^−1^ and PFOA concentrations of 32 ± 22 ng g^−1^. More recent studies have noted a change in the types of PFAS entering plants, with some transformation of different precursors to terminal metabolites during wastewater treatment, anaerobic digestion, or composting of biosolids. A survey of urban residuals in France noted that while PFOS and PFOA were frequently detected, their concentrations were lower than those reported by Venkatesan and Halden (2013) (Munoz et al., 2022). PFOS in that study ranged from 0.4 to 284 ng g^−1^ (*n* = 10). The highest PFOA concentration in this study was 13 ng g^−1^ (Munoz et al., 2022). In their place the authors identified higher concentrations of electrochemical fluorination‐sulfonamide precursors such as *N*‐methyl perfluorooctane sulfonamidoacetic acid (MeFOSAA), X:3 fluorotelomer carboxylates, and X:2 fluorotelomer sulfonates.

An extensive survey of biosolids in Michigan (biosolids from 181 treatment plants) found that PFOS (96%) and PFOA (63%) were present in a majority of the materials tested (Link et al., 2024). Mean and median concentrations of PFOS (40 ± 179, 10 ng g^−1^) and PFOA (4.8 ± 11, 1.2 ng g^−1^) were lower than reported from the analysis of 2001 biosolids. The authors also detected emerging PFAS compounds in a majority of the samples including perfluorohexanoic acid (PFHxA) (76% of samples), MeFOSAA (92%), and *N*‐ethyl FOSAA (87%). A survey conducted on influent, effluent, and biosolids from 27 treatment plants in Canada found high occurrences of precursors in influent with much lower concentrations of longer chain compounds (Gewurtz et al., 2024). Frequency of detection patterns for the different PFAS in biosolids were similar to those in the Michigan study. These results were also echoed by a sampling done of biosolids in Australia (Moodie et al., 2021). This survey also found high concentrations of polyfluoroalkyl phosphate diesters in the biosolids, another kind of precursor. The authors noted that the mean annual per person contribution of PFAS to wastewater was 6 mg (sum of 44 PFAS compounds). The results of these studies suggest that PFAS are ubiquitous in municipal wastewater, likely as a consequence of wide scale use of perfluorinated compounds in a broad range of everyday products (Glüge et al., 2020).

The US EPA recently conducted a draft risk assessment on PFAS in biosolids with human health as a focus (US EPA, 2025). The authors here used 1 n g^−1^ of PFOA and PFOS for this estimate. The focus of the draft risk assessment was a farm family. They modeled farms with high annual loading rates (10 Mg ha^−1^) for a 40‐year period. Exposure pathways included meat and fish consumption, drinking water, and consumption of plants grown in the biosolids‐amended soils. The farm family modeled consumed most of their foods from the farm and drank water (1 L per day) from a well, placed up to 2 m below the soil surface. The draft assessment found significant risk for select exposure pathways based on limited data and highly conservative assumptions (US EPA, 2025). The study did not evaluate risk to the general public from PFAS in biosolids applications (US EPA, 2025). The concentrations of PFOS used as a baseline are at the very low end for non‐industrially contaminated biosolids. These values are below concentrations in biosolids that come from homes and other non‐industrial sources. For example, a study found PFOA concentrations (median 0.85 µg kg^−1^ and mean 2.63 µg kg^−1^) and PFOS (median 12.6 µg kg^−1^ and mean 13.8 µg kg^−1^) concentrations in biosolids with no significant industrial input (Lin et al., 2024). The PFAS in these biosolids comes primarily from domestic sources, suggesting that daily home exposure may be a much more significant pathway than exposure through land application of biosolids.

Drinking water, food, food packaging, personal care products, and indoor dust are all potential pathways for home exposure to PFAS. PFAS in household dust has been quantified globally, with total median concentrations ranging from 17.3 to 197 ng g^−1^ (Tan et al., 2024). Gustafsson et al. (2022) estimated that dust inhalation by children was 3.0 ng kg^−1^ body weight (bw) per day and that this exposure route was close to the established weekly intake limit set by the European Food Safety Authority. Dietary exposure has also been noted as a significant source of PFAS (Domingo & Nadal, 2017; Tittlemier et al., 2006). Shan et al. (2016) estimated daily intake of PFOA and PFOS through diet 11.95 and 50 ng kg^−1^ bw but concluded that estimated daily PFAS exposure was lower than the suggested acceptable daily intake (PFOS 150 ng kg^−1^ bw day; PFOA 1500 ng kg^−1^ bw day). Consumption of fast foods and popcorn has also been associated with elevated PFAS (Hampson et al., 2024; Susmann et al., 2019). Detection of PFAS in drinking water is widespread with one study estimating that 18–80 million people in the US have PFOA and PFOS concentrations of 10 ng L^−1^ or higher in their drinking water (Andrews & Naidenko, 2020). Elevated concentrations of PFAS have been reported in a range of personal care products, suggesting additional exposure potential (Boronow et al., 2019; Whitehead et al., 2021).

In addition to phasing out the use of PFOA and PFOS, additional measures have been proposed or enacted to reduce the amount of PFAS entering the environment (National Caucus of Environmental Legislators, 2025). Several states have adopted measures to reduce the potential for excessive PFAS to enter soil systems through land application of biosolids. Michigan categorized biosolids based on their PFAS concentrations (Michigan; Minnesota Pollution Control Agency, n.d.). In Michigan, biosolids with PFOS or PFOA concentrations greater than 100 ng g^−1^ show clear signs of industrial contamination and are prohibited from land application. Biosolids with PFOA or PFOS concentrations less than 20 ng g^−1^ show no signs of significant industrial contamination with the primary source of these compounds being domestic flow and are eligible for land application based on general permit requirements.

Washington State has promoted the beneficial use of biosolids for several decades. Biosolids are used for a wide range of crops in the state including dryland wheat and forest fertilization as well as for garden vegetables. However, it is not clear that the assumptions used in the risk assessment draft are applicable to biosolids end uses within the state. To assess whether PFAS concentrations in biosolids pose a significant pathway for human exposure, the Washington State Department of Ecology (WA DOE) conducted a voluntary testing of biosolids across the state. Results from the survey are presented. Here the survey results are put into the context of biosolids use across the state. We consider the potential for excess human exposure through the different pathways that were considered in the EPA draft in the context of end uses in Washington. This was done to determine whether land application of biosolids has the potential to pose a significant pathway to PFAS exposure for state residents. This is an alternative approach to the hypothetical farm family modeled in the recent EPA draft risk assessment and is meant as a real‐world comparison to that exercise.

**Core Ideas**

- Per‐ and poly‐fluorinated alkyl substances (PFAS) concentrations in 44 biosolids in Washington State were generally similar to those reported at water recovery facilities in Michigan, suggesting that there will be measurable concentrations of PFAS in a majority of biosolids.
- The total oxidizable precursor assay did not reliably predict individual PFAS concentrations as measured by the Environmental Protection Agency 1633 protocol.
- End use of biosolids in Washington State suggests minimal risk for identification of a highly exposed individual for the majority of beneficial uses.

## 2 MATERIALS AND METHODS

### 2.1 PFAS sampling and analysis

The WA DOE offered voluntary testing of municipal biosolids. Forty‐four of approximately 300 wastewater treatment facilities in the state participated in the testing. The treatment plants sampled included larger facilities (*n* = 33) and smaller facilities (*n* = 11) from across the state (Sassi et al., 2025). Larger is defined as facilities that have daily influent of more than 1,000,000 gallons with smaller facilities receiving less than that amount. Participants included 13 facilities producing less than 299 dry tons of biosolids annually, 14 producing between 200 and 1499 dry tons of biosolids annually and 10 plants generating more than 1500 dry tons per year (Emily Kijowski, WA DOE, personal communication, 2025). Biosolids samples were collected by WA DOE staff in the spring of 2024 using the same protocol as had been used in Michigan (Michigan DEQ, 2022; Sassi, 2024; Sassi et al., 2025). Samples ranged from liquid (solids concentrations < 5%) to dry materials (solids concentrations > 90%). Liquid and solid fractions of the samples were not separated prior to analysis as each sample consisted of a composite of at least 10 grab samples with three samples collected per facility. Samples were put into PFAS‐free containers with HDPE or polypropylene caps and cooled to 0°C–6°C prior to shipment. At the lab, samples were kept no longer than 90 days and stored in the dark at or below −20°C prior to analysis. Triplicate samples were analyzed for each facility. A field blank and equipment blank were included for each triplicate set of samples. Matrix spiked samples as well as duplicate matrix spike samples were run in every analysis of up to 20 biosolids samples. Biosolids samples from seven different plants were used as laboratory duplicates, in addition to the triplicate samples from each treatment plant. As stated in the final report, all equipment blanks and all but one field blank were below detection limits. The field blank that indicated contamination had negligible concentrations of PFAS (<0.008 ng g^−1^). All samples were analyzed for PFAS by the SGS AXYS Laboratory using EPA Method 1633 (US EPA, 2024). A subset of samples was also analyzed at the same laboratory for total oxidizable precursors (TOP). The method detection limit for EPA 1633 ranged from 0.04 to 0.87 ng g^−1^, for the TOP assay 0.2–5 ng g^−1^. Additional details on biosolids sampling and analysis are available in the Quality Assurance plan for this project as well as in the final report (Sassi, 2024; Sassi et al., 2025). Biosolids with solids concentrations of less than 5% analyzed using Method 1633 showed very high variability across samples (>100%) in comparison to biosolids with higher percent solids. These are also not representative of the dewatered “finished product” biosolids land applied in Washington. Therefore, the results from those samples have been excluded. For the dewatered biosolids, results here include nine from smaller facilities and 29 from major facilities.

### 2.2 Biosolids end use

The majority of the biosolids generated in Washington State meet Class B standards for beneficial use and are applied to a range of agronomic and forestry crops (Figure 1a,b). Data from the 2020 and 2021 permitting process were used to determine end use for the biosolids generated in the state (Emily Kijowski, WA DOE, personal communication, 2024). A significant percentage of the biosolids are treated to meet Class A or Exceptional Quality standards and so are suitable for general sale and/or distribution in the state. A fraction of the biosolids is disposed of through combustion. All Class B biosolids require permits prior to land application. The permits specify application location, application rate, and crop to be grown. The biosolids application rate is determined for each end use and field based on soil nitrate. Application rates for dryland wheat are typically between 2 and 4 dry tons per acre with fields being reapplied every 4 years (Cogger et al., 2013). Forest application rates range from 3 to 7 dry tons per acre with a typical stand receiving up to five applications over a 40‐year period (Leonard et al., 2024). Vegetables planted in community gardens are often grown in raised beds or containers that have been filled with Class A biosolids‐based potting soils (Niedzwiecki et al., 2022; Una et al., 2022).

**FIGURE 1:**
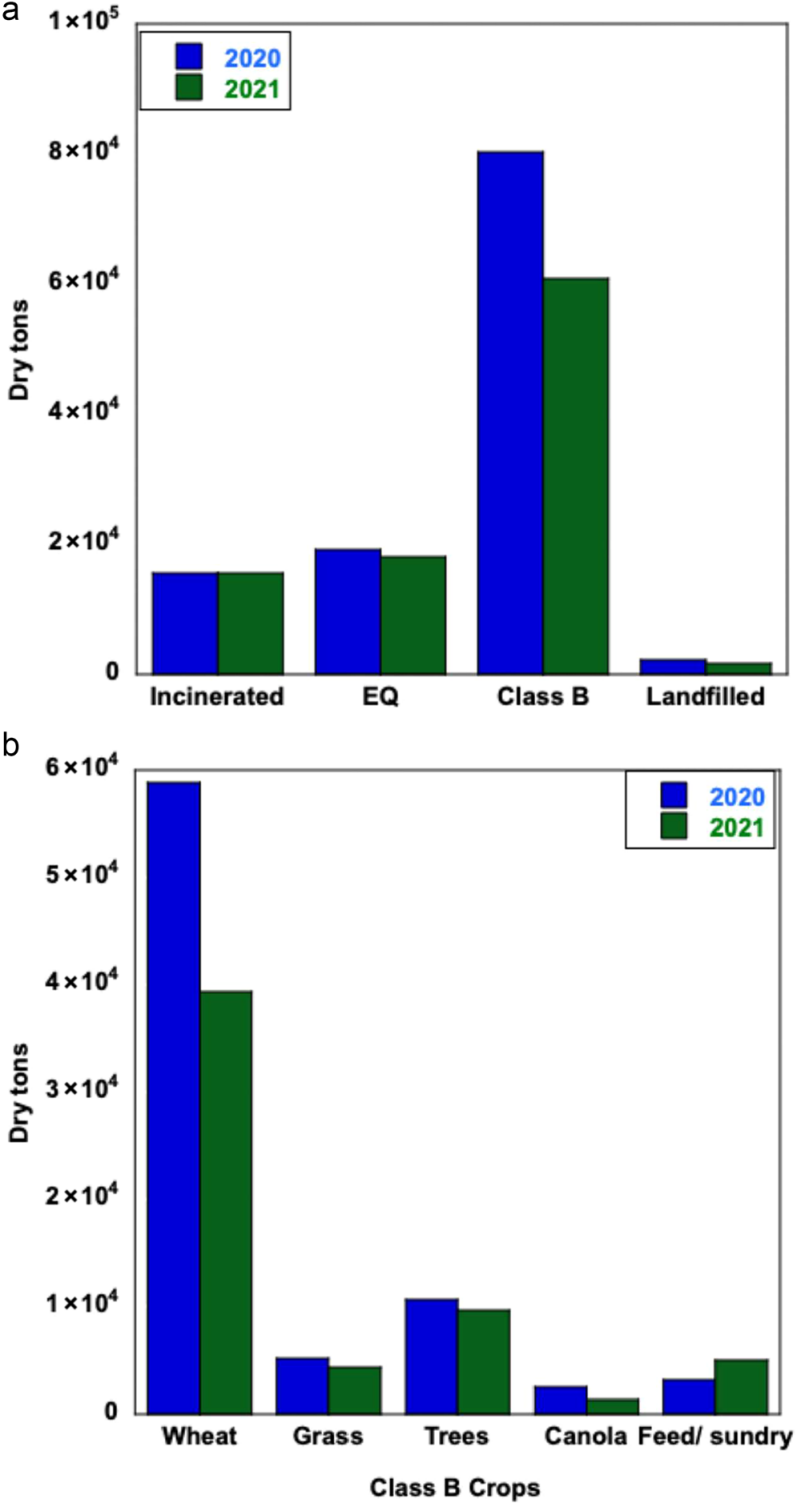
(a) Distribution of end use/disposal for biosolids produced in Washington State in 2020–2021. (b) End use of Class B biosolids in Washington State as specified in permit applications that are required for beneficial use. EQ stands for exceptional quality and signifies biosolids suitable for general home use that do not require a permit from the Department of Ecology.

### 2.3 Exposure/risk potential

We separated end use based on crop type. The majority of the Class B biosolids in the state are used to fertilize dryland wheat (73% in 2020 and 66% in 2021) with commercial forestry (13% in 2020 and 16% in 2021) as the next largest use (Emily Kijowski, WA DOE, personal communication, 2024). Grass for hay accounted for between 6% and 7% of the biosolids end uses. Smaller portions are used to fertilize canola (2%–3%) and sundry uses including crops for animal feed (4%–8%). The distribution for each year, based on crop type, is shown in Figure 1b. Class A biosolids can be distributed to the general public for any number of uses, so information on end use was not available. However, Tacoma, WA, is the largest municipality that produces Class A biosolids in the state. Their biosolids are blended with other materials to produce different soil products. A portion of the potting soil, produced with biosolids, sawdust, and aged wood, is donated to Harvest Pierce County, a regional program supporting community gardens and urban farms (Niedzwiecki et al., 2022). We used the Harvest Pierce County program to model the potential for PFAS transfer through the food chain from use of Class A biosolids. As would be typical for Class A use in community gardens in urban areas, drinking water is provided by residents by the municipality. Because of this, groundwater exposure through home wells is not pertinent. To estimate potential exposure through plant uptake, we placed the biosolids PFAS concentrations from the survey in the context of the published literature on plant uptake of different PFAS compounds for the crops grown with biosolids fertilization.

This exercise is not designed as a traditional risk assessment. Traditional risk assessment has focused on a highly exposed individual (HEI), such as a farmer who drinks well water and eats primarily homegrown vegetables in cases where high rates of biosolids have been applied. (US EPA, 2025). There is insufficient data on each of the exposure pathways for non‐industrially contaminated biosolids as they are used in Washington State. Here, we use the basic approach of a risk assessment—including identification of pertinent exposure pathways (movement to groundwater and uptake into plant tissue) to evaluate which end uses (if any) of biosolids in Washington State merit additional investigation.

## 3 RESULTS AND DISCUSSION

### 3.1 PFAS concentrations in Washington biosolids

PFAS were identified in all the biosolids sampled as part of the voluntary survey. Detection rates, mean, and median concentrations, as well as standard deviations are shown in Table 1. None of the utilities had PFOA concentrations greater than 20 ng g^−1^. Ten had mean PFOS concentrations greater than 20 ng g^−1^, with the highest reported mean concentration from a single utility of 59.3 ng g^−1^. Six of the utilities with PFOS concentrations >20 ng g^−1^ produced less than 299 dry tons of biosolids per year. The remaining four utilities with biosolids PFOS concentrations >20 ng g^−1^ were evenly distributed between medium‐sized and large facilities. Using the Michigan standards as a guideline, these results suggest that the biosolids from 27 of the participating utilities had domestic flow as their primary source of PFOA and PFOS. Ten showed elevated concentrations of PFOS, suggesting some level of industrial input. Based on the Michigan standards, none of the municipalities that participated in the voluntary testing had high enough levels of PFOS or PFOA to prohibit land application.

**TABLE 1:** Results from the WA DOE voluntary PFAS sampling.

		Detection rate (%)	ML	Mean (ng g)	Median (ng g)	SD
**PFSA (perfluorosulfonic acids)**						
Long chain						
	PFDS (perfluorodecanesulfonic acid)	51	0.2	0.77	0.41	1.25
	PFNS (perfluorononanesulfonic acid)	0	0.2			
	PFOS (perfluorooctane sulfonic acid)	100	0.2	18.95	13.8	15.2
	PFHpS (perfluoroheptanesulfonic acid)	8	0.2	0.12	0.1	0.08
	PFHxS (perfluorohexanesulfonic acid)	68	0.2	1.1	0.69	2.47
Short chain						
	PFPeS (perfluoropentansulfonic acid)	0	0.2			
	PFBS (perfluorobutanesulfonic acid)	41	0.2	0.79	0.1	1.61
**PFCA (perfluoroalkyl carboxylic acids)**						
Long chain						
	PFTeDA (perfluorotetradecanoic acid)	84	0.2	0.69	0.66	0.56
	PFTrDA (perfluorotridecanoic acid)	76	0.2	0.7	0.685	0.71
	PFDoDA (perfluorododecanoic acid)	97	0.2	1.71	1.34	1.45
	PFUnDA (perfluoroundecanoic acid)	97	0.2	1.15	0.82	1.29
	PFDA (perfluorodecanoic acid)	100	0.2	4.63	3.22	4.86
	PFNA (perfluorononanoic acid)	86	0.2	1.06	0.93	0.81
	PFOA (perfluorooctanoic acid)	68	0.2	2.84	1.86	3.74
Short chain						
	PFHpA (perfluoroheptanoic acid)	32	0.2	0.33	0.1	0.58
	PFHxA (perfluorohexanoic acid)	84	0.2	3.23	1.41	5.9
	PFPeA (perfluoropentanoic acid)	49	0.2	2.1	0.2	3.46
	PFBA (perfluorobutanoic acid)	19	0.2	0.77	0.4	1.19
Sulfonamide						
	MeFOSAA (methyl perfluorooctanesulfonamidoacetic acid)	100	0.2	9.53	9.55	7.9
	*N*‐MeFOSAA (N‐methyl perfluorooctanesulfonamidoacetic acid)	65	0.2	9.5	9.5	7.9
	EtFOSAA (ethyl perfluorooctanesulfonamidoacetic acid)	97	0.2	4.14	3.83	2.43
	FOSAA (perfluorooctanesulfonamidoacetic acid)	76	0.2	1.8	1.17	1.99
Fluorotelomer						
	8:2 FTSA (8:2 fluorotelomer sulfonic acid)	76	0.8	2.78	0.4	11.3
	6:2 FTSA (6:2 fluorotelomer sulfonic acid)	3	0.8	0.52	0.4	0.73
	5:3 FTCA (2H,2H,3H,3H‐perfluorooctanoic acid)	95	5	51.7	40.6	43
	4:2 FTSA (4:2 fluorotelomer sulfonic acid)	0	0.8			

*Note*: Samples were tested using Environmental Protection Agency method 1633. An additional 16 compounds were analyzed beyond those analyzed in the Michigan study. Of those, only those detected in the biosolids are included in the table. PFOA ranged from below detection to 18.8, and PFOS ranged from 0.5 to 59.3 ng g^−1^. For some compounds the computed mean is lower than the ML due to a large fraction of the samples being below this value and half the ML being used for the purpose of computing the statistics.

Abbreviations: EtFOSAA, ethyl FOSA; FOSA, perfluorooctane sulfonamidoacetic acid; MeFOSAA, methyl FOSA; ML, minimum level of quantification; PFAS, per‐ and poly‐fluorinated alkyl substances; PFBS, perfluorobutane sulfonate; PFHxA, perfluorohexanoic acid; PFHxS, perfluorohexane sulfonate; PFOA, perfluorooctanoic acid; PFOS, perfluorooctane sulfonic acid; WA DOE, Washington State Department of Ecology.

The results from this sampling were compared with the results from the Michigan sampling (Figure 2) (Link et al., 2024). Note that the Michigan study analyzed 24 compounds because it was conducted before EPA Method 1633, which includes analysis of 40 compounds. Both detection frequencies and mean concentrations from the two states are shown. In general, detection rates of PFOA and PFOS were similar across both states. Biosolids in Washington had generally higher detection rates of long‐chain PFCAs and fluorotelomers than the biosolids in Michigan. This may be a consequence of improved analytical techniques rather than differences in biosolids characteristics. Lower detection limits in the latest method could lead to higher detection frequencies. For these compounds, below detection values were reported as half of the reporting limit. While mean concentrations of PFOA were similar in both states, the mean PFOS concentration in Michigan biosolids was about double the measured concentrations in Washington biosolids. Concentrations of all other measured PFAS were generally similar. This comparison suggests that municipal biosolids will contain a range of PFAS compounds that are most likely reflective of both domestic and industrial flows (Link et al., 2024).

**FIGURE 2:**
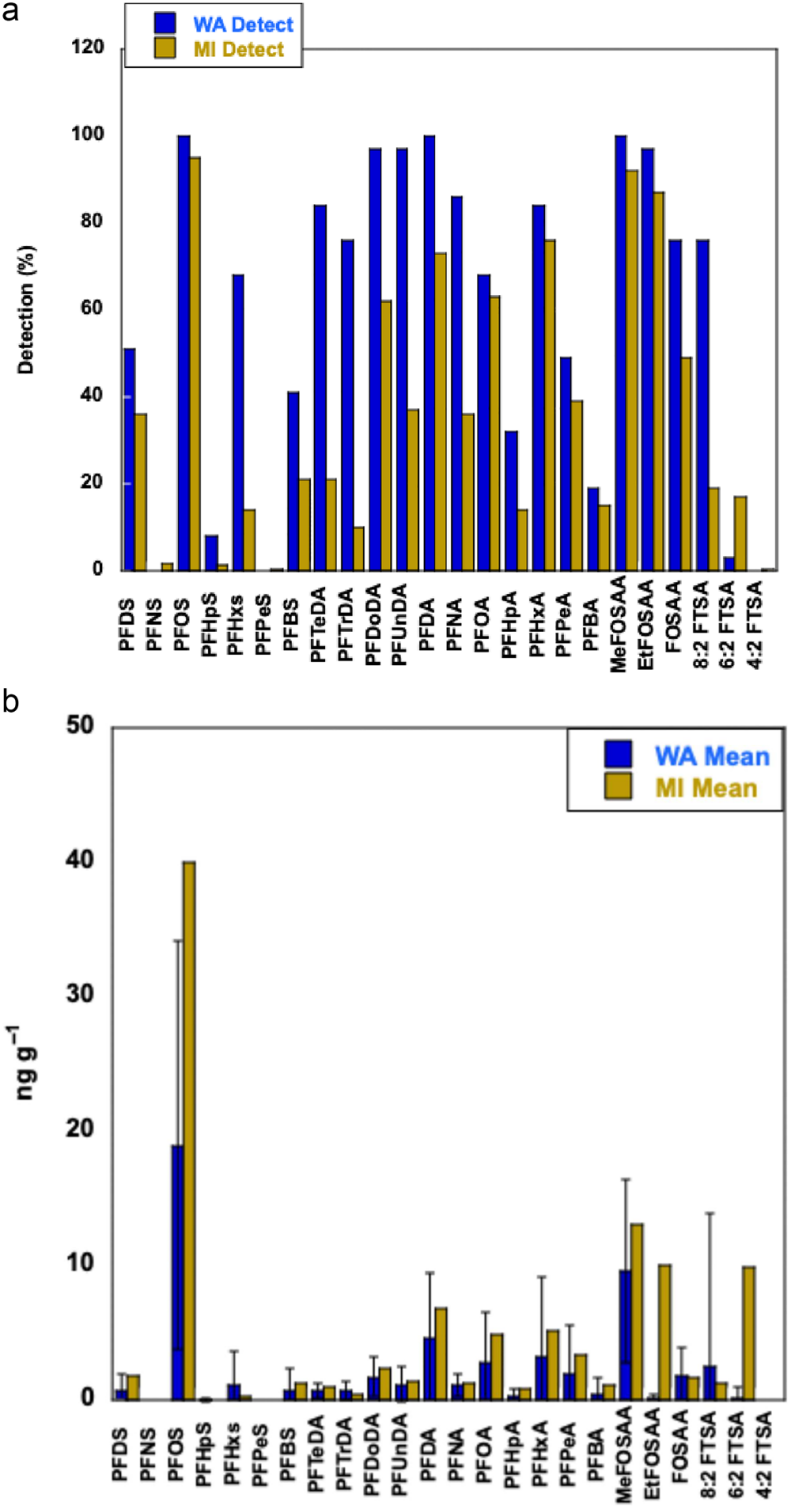
A comparison of per‐ and poly‐fluorinated alkyl substances (PFAS) results from the voluntary sampling in Washington State (WA) and the mandatory sampling in Michigan (MI). Figure 2a shows the detection percentage of the measured PFAS compounds across the biosolids measured in the two states. Figure 2b shows the mean and standard error concentrations of the different PFAS compounds measured. EtFOSA, ethyl; FOSA, perfluorooctane sulfonamidoacetic acid; MeFOSAA, methyl FOSA; PFBS, perfluorobutane sulfonate; PFHxA, perfluorohexanoic acid; PFHxS, perfluorohexane sulfonate; PFOA, perfluorooctanoic acid; PFOS, perfluorooctane sulfonic acid.

### 3.2 TOP assay

A subset of the municipalities also tested their biosolids using the TOP assay (Table 2). This assay has the potential to quantify PFAS precursors and the wide range of PFAS currently in everyday use (Baqar et al., 2024). The TOP assay uses Method 1633 for analysis, but quantifies PFAA precursors by putting a split of the sample through a digestion step to oxidize the precursors into forms measurable by 1633 and comparing the concentrations to the undigested sample. This provides a good estimate of the precursors present, but it may not fully quantify all precursors present because not all would necessarily be transformed to measurable types of PFAS during digestion. A number of studies have noted transformation of PFAS during wastewater treatment (e.g., Alukkal et al., 2024; Helmer et al., 2022). As a result of microbial transformations and/or volatile solids reductions, concentrations of longer chain compounds are often higher in municipal biosolids than would be predicted based on wastewater influent. Lazcano et al. (2020) used the TOP assay in addition to EPA 1633 for analysis of commercially available biosolids products. They found that the total PFCA concentration measured was greater after digestion, indicating the presence of PFAS precursors. The same result was observed in this study. However, the concentration of PFOA measured using EPA 1633 and that measured using the TOP assay varied significantly. This suggests very different PFAS precursor profiles across the treatment plants. The TOP assay is not a reliable predictor of formation of PFOA in the environment from precursors. The aggressive persulfate oxidation used to transform the precursors creates different transformation products than would be created under biotic or abiotic process in the environment (Ateia et al., 2023) and is limited to producing only carboxylic acids. Therefore, while TOP is very useful in quantifying the overall level of precursors present in a sample, it cannot be used to predict the ultimate concentration of any individual PFAS compounds in the environment.

**TABLE 2:** PFOA and PFOS in selected biosolids samples from different wastewater treatment plants (WWTP) as measured using EPA 1633 and the TOP assay. The ratio of PFOA and PFOS measured using 1633 and the TOP assay is also shown for each sample.

WWTP	Total PFAS	TOP PFAS (ng g^−1^)	Measured PFOA (ng g^−1^)	TOP PFOA	Molar ratio PFOA TOP/measured
WWTP1	330	679	0.04	69.0	1725
WWTP3	236	662	0.04	96.4	2410
WWTP4	147	218	2.3	18.0	8
WWTP6	72	489	0.04	48.6	1215
WWTP7	223	543	1.96	44.3	23
WWTP8	66	484	0.04	43.7	1093
WWTP9	231	4218	0.04	795.0	19,875
WWTP10	104	1001	8.58	67.0	8
WWTP11	205	308	4.5	25.2	6
WWTP17	148	527	2.1	54.6	26
WWTP18	58	226	0.04	22.2	555
WWTP19	78	91	0.41	10.1	25
WWTP21	183	592	0.8	48.2	60
WWTP23	201	270	1.2	26.4	22
WWTP24	172	869	0.04	76.2	1905
WWTP26	177	439	3.98	47.3	12
WWTP27	127	536	0.68	63.7	94
WWTP30	71	101	0.04	4.5	114
WWTP32	140	739	0.92	56.0	61
WWTP34	143	520	0.04	48.8	1220
WWTP35	124	266	1.35	14.7	11
WWTP44	414	1066	15.7	84.8	5

Abbreviations: EPA, Environmental Protection Agency; PFAS, per‐ and poly‐fluorinated alkyl substances; PFOA, perfluorooctanoic acid; PFOS, perfluorooctane sulfonic acid; TOP, total oxidizable precursors.

### 3.3 Risk potential

Washington State has recently ruled to require PFAS testing for all biosolids (https://ecology.wa.gov/waste‐toxics/reducing‐recycling‐waste/biosolids/senate‐bill‐5033). It is not clear if the state will require pretreatment for instances that suggest the potential for industrial input. If the state were to enact pretreatment, the results from the draft EPA risk assessment suggest that there would still be concerns for the EPA‐modeled farm family. However, the way that biosolids are used in Washington State is not represented by the exposure scenarios used by EPA in their exercise (Table 3). To evaluate the potential for exposure, we considered each of the biosolids end uses identified separately. Exposure pathways for each end use were evaluated based on available literature and state‐specific conditions. The pathways considered include drinking water through movement to groundwater and plant uptake for direct human or animal consumption.

**TABLE 3:** Increased cancer risk factors for an 80 kg adult living on a farm with biosolids‐amended soil (400 Mt ha) for a 10‐year period used by EPA for PFOA risk assessment and comparison to biosolids uses in Washington State.

Pathway	Assumptions	Increased cancer risk
EPA		PFOA
Biosolids soil groundwater	1 L water a day for 10 years	5.4 × 10^−4^
Biosolids soil surface water	1 L water a day for 10 years	5.4 × 10^−4^
Biosolids soil surface water fish	Consumption of 9.24 ounces of fish per week for 10 years	6.3 × 10^−4^
Biosolids soil fruit/vegetables	0.5–1 Serving per day 100–200 g for 10 years	2.2 × 10^−5^–6.3 × 10^−6^
Biosolids soil cow beef	One 3 ounce serving per day for 10 years	3.4 × 10^−5^
Biosolids soil cow milk	32 ounces of milk per day for 10 years	4.1 × 10^−4^
Biosolids soil chicken poultry	One 3 ounce serving per day for 10 years	2.8 × 10^−6^
Biosolids soil chicken egg	1 egg per day for 10 years	7.7 × 10^−5^

Washington State		
	Time to cumulative loading used by EPA	Pertinent risk pathway
Dryland wheat	238 Years	None
Tree plantations	400 Years	None
Canola	60 (Assuming annual applications of 3 tons per acre)	Ground or surface water
Grass/animal feed	60	Ground or surface water, beef
Class A vegetables/fruit	20 Years (assuming mix is 20% biosolids by dry weight and mix is applied at 100 Mt ha)	Vegetable fruit consumption

Abbreviations: EPA, Environmental Protection Agency; PFOA, perfluorooctanoic acid.

### 3.4 Dryland wheat

The primary type of wheat grown in the state is dryland soft wheat (*Triticum aestivum*). In 2024, 2,146,415 acres were planted in winter wheat (https://wagrains.org/wp‐content/uploads/2024/09/2024‐WA‐Wheat‐Variety‐Survey‐CORRECTED.pdf). Biosolids applications are centered in Douglas County with yearly applications to approximately 12,000 acres (Brown et al., 2026; USDA, 2017). The City of Spokane also applies biosolids to about 3000 acres of dryland wheat annually. Biosolids are typically applied once every 4 years at a rate of between 2 and 4 dry tons per acre (Brown et al., 2026) (https://my.spokanecity.org/publicworks/wastewater/biosolids/). The primary exposure pathway is direct consumption of wheat grain. A number of studies have looked at PFAS uptake into grain tissue for various grass species. Yang et al. (2024) measured PFAS concentrations in soils and wheat grain from ten cities in China with no history of biosolids use. Total PFAS were quantified in soils and grain sampled with soil concentrations ranging from 0.34 to 1.59 ng g^−1^ and grain concentrations ranging from 2.74 to 6.01 ng g^−1^. PFOS and PFOA made up 15.6% and 23% of the total soil PFAS. While PFOS was below detection in wheat grain, PFOA was detected in all samples, with 26% of the PFAS in grain tissue being PFOA. Despite a bioaccumulation factor (BAF) of 5.14, the authors concluded that PFAS in wheat grain as a component of a typical diet does not pose a significant exposure risk as a result of a low risk quotient. Another study of PFAS in wheat grown on high loading rates of biosolids showed very low BAF for both PFOA and PFOS in wheat grain (Wen et al., 2014). Soil PFOA in this study ranged from 0.61 ng g^−1^ (Control) to 26.1 ng g^−1^ with PFOS ranging from 0.61 to 40.8 ng g^−1^. Grain concentrations of PFOA ranged from below detection limits in the control soil to 2.9 ± 0.2 ng g^−1^ in the highest rate. PFOS concentrations ranged from below detection to 2.53 ± 0.21 ng g^−1^. These two studies suggest that PFAS uptake in plants does not increase with increased soil PFAS. Two other studies found PFAS concentrations in grain tissue for oats and corn below detection levels for crops grown in biosolids‐amended soils (Black et al., 2025; Blaine et al., 2013).

Both research findings and standard farming practices suggest that plant uptake of PFAS into wheat grain is not a significant cause for concern in Washington State. The initial survey shows results, that even without a requirement for industrial pretreatment, would require centuries of application to reach soil PFAS concentrations observed in the Wen et al. (2014) study. It is also not clear that domestically impacted PFAS biosolids would result in significant PFAS uptake into grain (Black et al., 2025; Blaine et al., 2013). Additionally, wheat is typically harvested and milled on a large scale, not by the home gardener. Wheat grown in biosolids‐amended soils is not separated from conventionally grown wheat at harvest. Soft wheat is a specialty wheat varietal typically used for baking. In 2018, soft wheat made up 16% of the total wheat consumed in the United States (https://www.ers.usda.gov/data‐products/wheat‐data/wheat‐data‐visualization/). Based on the low portion of the total wheat grown in the state that is fertilized with biosolids (15,000 acres versus over 2 million acres) and the type of wheat grown, it is highly unlikely, if not impossible, for a farm family to have elevated exposure to PFAS through ingesting wheat grown in biosolids‐amended soils.

Other exposure pathways considered by EPA include movement of PFAS to surface and groundwater used for drinking water. The areas used for dryland wheat cultivation are typically limited by insufficient precipitation. Average annual precipitation in Douglas County is 12 inches. The reported depth to groundwater is 325 feet (https://nwis.waterdata.usgs.gov/wa/nwis/gwlevels/?site_no=474346120023801&agency_cd=USGS&). Both of these factors suggest that exposure through drinking water is an unrealistic concern for areas where dryland wheat is grown.

### 3.5 Commercial tree plantations

Biosolids are used to enhance tree growth in commercial Douglas fir (*Pseudotsuga menziesii*) plantations (Leonard et al., 2024). King County has the largest program in the state applying biosolids to forest plantations with 9883 dry tons (8968 Mg) applied in 2020 and 9003 (8170 Mg) dry tons applied in 2021 (Emily Kijowski, WA DOE, personal communication, 2024). At an application rate of 4 dry tons per acre, that would suggest average applications to approximately 2500 acres each year. The biosolids are applied to commercial plantations that cover 65,000 ha, of which 7300 ha have been identified as suitable for biosolids application. Slope restrictions and proximity to surface water limit the area suitable for biosolids applications. The typical rotation in these plantations is 40 years with biosolids applied to meet the N needs of the stand up to five times during a rotation. The forests are accessible to the general public by permit for recreation but there are no homes located within the tree farms. As there are no food crops produced, the dietary exposure pathway does not apply. Movement to groundwater would be the only potential exposure pathway for this end use. With no residences on site, individual wells directly below biosolids applications are not a concern. Movement of PFAS to groundwater with transport offsite via flow within an aquifer would be the likeliest pathway for exposure. Previous work suggests limited potential for movement of PFOA and PFOS to groundwater directly below application sites, suggesting minimal potential for large‐scale contamination of an aquifer from infrequent biosolids application to a small portion of the forest.

Studies have shown that factors including soil texture, depth to groundwater, annual precipitation, and absorption at the air–water interface will all factor into the potential for longer chain PFAS to move through the soil profile (I. Pepper et al., 2023). In general, studies have seen attenuation of PFOA and PFOS with depth. For example, a nationwide survey of PFAS in biosolids‐amended soils found increasing soil attenuation with increasing chain length of different compounds (I. L. Pepper et al., 2026). Median soil concentrations of PFOA were 0.33 ng g at 30 cm, 0.05 ng g at 90 cm, and 0.02 ng g at 180 cm. Median concentrations of PFOS were 0.4 ng g at 30 cm, 0.04 ng g at 90 cm, and 0.01 ng g at 180 cm. A study looked at biosolids movement through soil at a long‐term dedicated disposal site (Alvarez‐Ruiz et al., 2024). Biosolids had been applied to this site annually for 38 years at a loading rate of 71 dry Mg ha^−1^. Soil sampling showed no movement of long‐chain PFAS below the 60‐cm depth. Concentrations of PFOA in groundwater samples from the four wells (1.8‐m depth) below the disposal site ranged from below detection (two wells) to 36.37 ng L^−1^ (one well) with concentration of 0.32 ng L^−1^ in the remaining well. Concentrations of PFOS ranged from 0.11 to 2.14 ng L^−1^. While short‐chain PFAS were elevated in one of the wells directly below the disposal site, they were generally close to or below detection in the other wells. These studies, along with the absence of drinking water wells on the site and the small portion of the area that has received biosolids, suggest no significant potential for movement to groundwater to be a concern at this site.

A prior exposure assessment focused on the forest hiker with drinking water from streams as the primary exposure pathway (Brown et al., 2019). Surface water would appear to be the only potential pathway of concern for human exposure. King County has monitored surface waters in the tree farms for several decades. A recent study showed no indication of movement of N to surface waters from biosolids applications (Leonard et al., 2024). Nitrogen is present in the biosolids at over 50,000 mg kg^−1^ with mineral forms highly soluble. Organic N would also move with soil particles via surface flow. We recognize that no evidence of N movement is not definitive proof that PFAS has not moved to surface waters; however, it does suggest limited transport of biosolids particles and biosolids‐borne constituents to surface waters. While PFAS in surface water has not been measured, low exposure frequency for the hiker combined with no evidence of N movement from the biosolids suggests that this end use poses minimal risk for human exposure to PFAS from the biosolids applications.

### 3.6 Class A—Urban use

The EPA draft risk assessment noted that consumption of fruits and vegetables grown on biosolids‐amended soils may present a risk for a farm family consuming homegrown vegetables. However, the draft assessment also noted that limited data are available. As a result, much of this aspect of the work was based on greenhouse studies and studies from industrially contaminated sites (US EPA, 2025). Home and community gardens are common in Washington State with the Master Gardener Program (https://mastergardener.wsu.edu/who‐we‐are/history/history‐of‐the‐master‐gardener‐program/) originating in the state.

Potential exposure to PFAS through consumption of homegrown vegetables is the most sensitive pathway for this example. A few studies have looked at uptake of PFAS into garden vegetables. Blaine et al. (2013) evaluated PFAS uptake by lettuce in biosolids‐amended soil in a field experiment. At the 100 Mg ha^−1^ application rate (which is 4x the typical agronomic rate), soil concentrations of PFOA and PFOS were 5.17 ± 0.28 and 13.91 ± 0.91 ng g^−1^, respectively. Concentrations of PFOA in lettuce were below quantification limits, with concentrations of PFOS of 1.39 ± 0.17 ng g^−1^. Tomato was also grown with concentrations below quantification limits for both compounds.

Sivaram et al. (2023) grew onions (*Allium cepa*) in a greenhouse trial using soils collected from fields that had received between 30 and 35 Mg ha^−1^ of biosolids. The concentrations of PFOA in the soils ranged from 0.93 to 3.19 ng g^−1^ and PFOS ranged from 3.43 to 48.07 ng g^−1^. Biosolids PFAS concentrations were not provided. The authors observed higher concentrations of PFOA in root tissue (0.55–1.82 ng g^−1^) than PFOS (0.03–0.13 ng g^−1^). However, they noted that eating the onions would not be a significant source of PFAS exposure, as intake of PFOA and PFOS did not exceed the recommended tolerable daily intake using Agency for Toxic Substances and Disease Registry consumption and toxicity values (ATSDR, 2005, 2021). This was the case even if one assumed that onions grown in the biosolids‐amended soil made up 100% of vegetable consumption. More recently, Dennis et al. (2025) tested uptake of a range of organic contaminants, including three PFAS compounds (perfluorobutane sulfonate, PFHxA, and perfluorohexane sulfonate), into spinach, radish, and broccoli tissue for field‐grown vegetables grown in Class A biosolids‐amended soils and irrigated with reclaimed water. None of the PFAS compounds were detected in the plant tissue.

The concentrations of PFOA and PFOS in the City of Tacoma's biosolids‐based potting soil (Tagro) have been reported as 11.4 ± 0.7 and 3.0 ± 0.4 ng g^−1^, well within the limits used in Michigan for unrestricted use (Kim Lazcano et al., 2020). These results suggest that while sampling vegetables grown with biosolids‐based soil amendments, such as those associated with the Harvest Pierce County program, would be recommended, there is a very low potential for those plants to contain sufficient PFAS to pose a realistic risk for human exposure.

### 3.7 Hay/animal feed/canola

In Washington State between 8000 and 9000 tons of biosolids were used for “grass” or “feed sundry” with an additional 1400–2500 tons used for canola in 2020 and 2021. The EPA draft risk assessment concluded that consuming animal products, including meat, milk, and eggs, from animals that had grazed on pastures with biosolids‐amended soils or consumed feed grown on those soils was a potential risk for a farm family whose primary source of meat was animals raised on the farm (US EPA, 2025). Two studies, both conducted on soils amended with industrially contaminated biosolids, identified significant uptake of PFOA and PFOS into grass tissue (Simones et al., 2024; Yoo et al., 2011). Simones et al. (2024) measured PFAS concentrations in grass mixtures grown on farms that had received biosolids contaminated with PFAS from paper manufacture. Samples were collected across multiple years at one farm (soil PFOS ranging from 44 to 232 ng g^−1^) and single‐year samples were collected from other farms. The bioconcentration factor (BCF) varied both within and across farms and was not consistent based on soil concentration. At the lowest soil PFOS concentrations (3.0 ± 0.4 to 19.1 ± 5.2 ng g^−1^) the BCF was 0.01. For comparison, the EPA draft risk assessment used a BCF of 0.25 for PFOA and 0.07 for PFOS (US EPA, 2025).

Another study measured PFAS concentrations in fescue with grasses harvested from a long‐term wastewater irrigation site, reflective of non‐contaminated effluent, and saw limited uptake of longer chain compounds (Mroczko et al., 2022). Uptake varied by grass harvest/cutting but total PFAS for the 20 compounds analyzed averaged 11.3 ng g^−1^. The authors estimated that total PFAS consumption for dairy cows, 42% of whose diet consists of crops grown on the site, ranged between 2.46 and 7.67 mg animal year^−1^. Two other studies of uptake by corn and oats, and also grass species, although not typically used for pastures, showed no uptake of PFOA or PFOS into leaf tissue or grain tissue for plants grown in non‐industrially impacted biosolids (Black et al., 2025; Blaine et al., 2013). These results suggest that application of non‐industrially impacted biosolids to pastures or for animal feed will have minimal potential for food chain transfer. Field sampling has the potential to provide additional confirmation.

A small portion of the biosolids generated in the state was used to grow canola. Canola can also be used for animal feed. Canola for meal is typically sent to processing facilities for finishing (Adewole et al., 2016). Meal from canola grown on the biosolids‐amended soils would be mixed with meal grown on conventionally fertilized soils, effectively diluting any potentially elevated PFAS concentrations. This practice, similar to wheat grain being sent off farm for finishing and sale, effectively reduces any potential for excess PFAS from biosolids in canola to impact animals consuming the meal. While direct grazing on canola has been examined in the literature, it is not a typical practice in Washington State (Lauriault et al., 2024). We were unable to find studies that looked at PFAS uptake into canola. Because direct grazing is not commonly practiced, it is unlikely that use of biosolids with PFAS concentrations characteristic of domestic sources would present a credible risk of heightened exposure.

For each of these end uses, there is a potential for movement to groundwater to be a concern. Peter and Lee (2025) measured surface and groundwater from sites in Indiana where biosolids had been applied. The authors found no indication of movement of PFAS from surface to groundwater, potentially as a result of an impermeable layer above the groundwater. Water from tile‐drained fields generally showed detectable but minimal impact from biosolids, with the exception of a site with very high cumulative loading rates. For application to grass, canola, or animal feed, site‐specific information, including cumulative loading rates, annual precipitation, depth to groundwater, and presence of wells, would be important to assess the potential for concern.

## 4 CONCLUSIONS

The results of the voluntary survey suggest that biosolids in Washington State, much like similar studies elsewhere, have detectable concentrations of a wide range of PFAS. As was shown, the PFAS concentrations here are comparable to what was reported for Michigan (Link et al., 2024). A portion of the biosolids tested show indications of industrial input. Requiring industrial pretreatment, as has been done in Michigan, would likely reduce the PFAS concentrations for those biosolids to less than 20 ppb for PFOA and PFOS (https://www.michigan.gov/egle/about/organization/water‐resources/biosolids/pfas‐related). These concentrations are reflective of domestic input, suggesting that home exposure is likely much more significant than any potential exposure via biosolids. Considering end uses for biosolids in Washington State suggests minimal potential human impact from use for dryland wheat and commercial tree plantations. Due to limited data for plant uptake and movement to groundwater for domestically impacted biosolids, additional sampling for the other end uses common in the state is recommended.

## AUTHOR CONTRIBUTIONS

**Sally Brown**: Conceptualization; formal analysis; investigation; writing—original draft. **Scott Mansell**: Data curation; formal analysis; writing—review and editing.

## CONFLICT OF INTEREST STATEMENT

The authors declare no conflicts of interest.

## References

[jeq270279-bib-0001] Adewole, D. I. , Rogiewicz, A. , Dyck, B. , & Slominski, B. A. (2016). Chemical and nutritive characteristics of canola meal from Canadian processing facilities. Animal Feed Science and Technology, 222, 17–30.

[jeq270279-bib-0002] Alukkal, C. R. , Lee, L. S. , & Staton, K. (2024). Per‐ and polyfluoroalkyl substances behavior: Insights from autothermal thermophilic aerobic digestion—Storage nitrification‐denitrification reactors. Chemosphere, 365, Article 143357. 10.1016/j.chemosphere.2024.143357 39293685

[jeq270279-bib-0003] Alvarez‐Ruiz, R. , Lee, L. S. , & Choi, Y. (2024). Fate of per‐ and polyfluoroalkyl substances at a 40‐year dedicated municipal biosolids land disposal site. Science of the Total Environment, 954, 176540. 10.1016/j.scitotenv.2024.176540 39332729

[jeq270279-bib-0004] Andrews, D. Q. , & Naidenko, O. V. (2020). Population‐wide exposure to per‐ and polyfluoroalkyl substances from drinking water in the United States. Environmental Science & Technology Letters, 7(12), 931–936.

[jeq270279-bib-0005] Ateia, M. , Chiang, D. , Cashman, M. , & Acheson, C. (2023). Total oxidizable precursor (TOP) assay—Best practices, capabilities and limitations for PFAS site investigation and remediation. Environmental Science & Technology Letters, 10(4), 292–301. 10.1021/acs.estlett.3c00061 PMC10259459 37313434

[jeq270279-bib-0006] ATSDR . (2005). Public health assessment guidance manual. US Department of Health and Human Services.

[jeq270279-bib-0007] ATSDR . (2021). Toxicological profile for perfluoroalkyls. US Department of Health and Human Services, Public Health Service.

[jeq270279-bib-0008] Baqar, M. , Chen, H. , Yao, Y. , & Sun, H. (2024). Latest trends in the environmental analysis of PFAS including nontarget analysis and EOF‐, AOF‐, and TOP‐based methodologies. Analytical and Bioanalytical Chemistry, 417, 555–571. 10.1007/s00216-024-05643-9 39570388

[jeq270279-bib-0009] Black, G. P. , Wong, L. , & Young, T. M. (2025). Uptake of per‐ and polyfluorinated alkyl substances by dry farmed oats following the agricultural application of biosolids and compost. Environmental Science: Processes & Impacts, 27(3), 661–669. 10.1039/d4em00502c PMC12017740 39907451

[jeq270279-bib-0010] Blaine, A. C. , Rich, C. D. , Hundal, L. S. , Lau, C. , Mills, M. A. , Harris, K. M. , & Higgins, C. P. (2013). Uptake of perfluoroalkyl acids into edible crops via land applied biosolids: Field and greenhouse studies. Environmental Science & Technology, 47, 14062–14069. https://pubs.acs.org/doi/10.1021/es403094q 10.1021/es403094q 24206563

[jeq270279-bib-0011] Boronow, K. E. , Brody, J. G. , Schaider, L. A. , Peaslee, G. F. , Havas, L. , & Cohn, B. A. (2019). Serum concentrations of PFASs and exposure‐related behaviors in African American and non‐Hispanic white women. Journal of Exposure Science & Environmental Epidemiology, 29, 206–217. 10.1038/s41370-018-0109-y PMC6380931 30622332

[jeq270279-bib-0012] Brown, S. , Kennedy, L. , Cullington, M. , Mihle, A. , & Lono‐Batura, M. (2019). Relating pharmaceuticals and personal‐care products in biosolids to home exposure. Urban Agriculture & Regional Food Systems, 4(1), 1–14. 10.2134/urbanag2018.12.0005

[jeq270279-bib-0013] Brown, S. , Vernik, M. , Butman, D. , Griffin‐LaHue, D. , & Finlinson, J. (2026). Quantifying the environmental and economic impact of municipal biosolids use in dryland wheat in Washington State. Urban Agriculture & Regional Food Systems, 11, e70038.

[jeq270279-bib-0014] Cogger, C. G. , Bary, A. I. , Kennedy, A. C. , & Fortuna, A.‐M. (2013). Long‐term crop and soil response to biosolids applications in dryland wheat. Journal of Environmental Quality, 42, 1872–1880. 10.2134/jeq2013.05.0109 25602427

[jeq270279-bib-0015] Dennis, N. M. , Braun, A. J. , Mafishiraz, Z. , & Gan, J. (2025). Investigation of organic contaminants in field‐cultivated vegetables receiving recycled water and biosolids applications. Environment International, 200, Article 109546. 10.1016/j.envint.2025.109546 40446753

[jeq270279-bib-0016] Domingo, J. L. , & Nadal, M. (2017). Per‐ and polyuoroalkyl substances (PFASs) in food and human dietary intake: A review of the recent scientific literature. Journal of Agricultural and Food Chemistry, 65, 533–543. 10.1021/acs.jafc.6b04683 28052194

[jeq270279-bib-0018] Gewurtz, S. B. , Auyeung, A. S. , De Silva, A. O. , Teslic, S. , & Smyth, S. A. (2024). Per‐ and polyfluoroalkyl substances (PFAS) in Canadian municipal wastewater and biosolids: Recent patterns and time trends 2009 to 2021. Science of the Total Environment, 912, Article 168638. 10.1016/j.scitotenv.2023.168638 37984658

[jeq270279-bib-0019] Ghaznavi, S. M. , Zimmerman, C. , Shea, M. E. , MacRae, J. D. , Pechenham, J. M. , Noblet, C. L. , Apul, O. G. , & Kopec, A. D. (2023). Management of per‐ and polyfluoroalkyl substances (PFAS)‐laden wastewater sludge in Maine: Perspectives on a wicked problem. Biointerphases, 18, 041004. 10.1116/6.0002796 37602771

[jeq270279-bib-0020] Glüge, J. , Scheringer, M. , Cousins, I. T. , DeWitt, J. C. , Goldenman, G. , Herzke, D. , Lohmann, R. , Ng, C. A. , & Wang, Z. (2020). An overview of the uses of per‐ and polyfluoroalkyl substances (PFAS). Environmental Science: Processes & Impacts, 22, 2345–2373. 10.1039/D0EM00291G PMC7784712 33125022

[jeq270279-bib-0021] Gustafsson, Å. , Bergman, Å. , & Weiss, J. M. (2022). Estimated daily intake of per‐ and polyfluoroalkyl substances related to different particle size fractions of house dust. Chemosphere, 303, 135061. 10.1016/j.chemosphere.2022.135061 35649447

[jeq270279-bib-0022] Hampson, H. E. , Costello, E. , Walker, D. I. , Wang, H. , Baumert, B. O. , Valvi, D. , Rock, S. , Jones, D. P. , Goran, M. I. , Gilliland, F. D. , Conti, D. V. , Alderete, T. L. , Chen, Z. , Chatzi, L. , & Goodrich, J. A. (2024). Associations of dietary intake and longitudinal measures of per‐ and polyfluoroalkyl substances (PFAS) in predominantly Hispanic young Adults: A multicohort study. Environment International, 185, Article 108454. 10.1016/j.envint.2024.108454 PMC11089812 38316574

[jeq270279-bib-0023] Helmer, R. W. , Reeves, D. M. , & Cassidy, D. P. (2022). Per‐ and polyfluorinated alkyl substances (PFAS) cycling within Michigan: Contaminated sites, landfills and wastewater treatment plants. Water Research, 210, 117983. 10.1016/j.watres.2021.117983 34954365

[jeq270279-bib-0024] Lazcano, K. R. , Choi, Y. J. , Mashtare, M. L. , & Lee, L. S. (2020). Characterizing and comparing per‐ and polyfluoroalkyl substances in commercially available biosolid and organic non‐biosolid‐based products. Environmental Science & Technology, 54, 8640–8648. 10.1021/acs.est.9b07281 32567309

[jeq270279-bib-0025] Lauriault, L. M. , Angadi, S. V. , Duff, G. C. , Scholljegerdes, E. J. , Darapuneni, M. K. , & Martinez, G. K. (2024). Influence of grazing on canola grain, canola forage yield, and beef cattle performance. Animals, 14, 371. 10.3390/ani14030371 PMC10854679 38338014

[jeq270279-bib-0026] Leonard, E. , Axt, B. , Brown, S. , & Butman, D. (2024). Impact of long‐term application of biosolids to surface water nitrogen in a working forest plantation in King County, Washington. Journal of Environmental Management, 360, Article 121184. 10.1016/j.jenvman.2024.121184 38796868

[jeq270279-bib-0027] Lin, D. , Méndez, M. , Paterson, K. , Wong, A. , Yee, D. , Sutton, R. , Houtz, E. , Cousins, M. , & Fono, L. (2024). Residential wastewater as a major source of per‐ and polyfluoroalkyl substances to municipal wastewater. ACS ES&T Water, 4, 4847–4857.

[jeq270279-bib-0028] Link, G. W. , Reeves, D. M. , Cassidy, D. P. , & Coffin, E. S. (2024). Per‐ and polyfluoroalkyl substances (PFAS) in final treated solids (biosolids) from 190 Michigan wastewater treatment plants. Journal of Hazardous Materials, 463, 132734. 10.1016/j.jhazmat.2023.132734 37922581

[jeq270279-bib-0030] Michigan DEQ . (2022). Biosolids and sludge PFAS sampling guidance . Michigan Department of Environment, Great lakes, and Energy. https://mimfg.org/Portals/0/Documents/GA/pfas_biosolids‐sludge‐sampling‐guidance_191020.pdf

[jeq270279-bib-0031] Minnesota Pollution Control Agency . (n.d.). PFAS in biosolids strategy . https://www.pca.state.mn.us/air‐water‐land‐climate/pfas‐in‐biosolids‐strategy

[jeq270279-bib-0032] Moodie, D. , Coggan, T. , Berry, K. , Kolobaric, A. , Fernandes, M. , Lee, E. , Reichman, S. , Nugegoda, D. , & Clarke, B. O. (2021). Legacy and emerging per‐ and polyfluoroalkyl substances (PFASs) in Australian biosolids. Chemosphere, 270, Article 129143. 10.1016/j.chemosphere.2020.129143 33429237

[jeq270279-bib-0033] Mroczko, O. , Preisendanz, H. E. , Wilson, C. , Mashtare, M. L. , Elliott, H. A. , Veith, T. L. , Soder, K. J. , & Watson, J. E. (2022). Spatiotemporal patterns of PFAS in water and crop tissue at a beneficial wastewater reuse site in central Pennsylvania. Journal of Environmental Quality, 51, 1282–1297. 10.1002/jeq2.20408 PMC9828414 36070520

[jeq270279-bib-0034] Munoz, G. , Michaud, A. M. , Liu, M. , Vo Duy, S. , Montenach, D. , Resseguier, C. , Watteau, F. , Sappin‐Didier, V. , Feder, F. , Morvan, T. , Houot, S. , Desrosiers, M. , Liu, J. , & Sauvé, S. (2022). Target and nontarget screening of PFAS in biosolids, composts, and other organic waste products for land application in France. Environmental Science & Technology, 56, 6056–6068. 10.1021/acs.est.1c03697 34668380

[jeq270279-bib-0035] National Caucus of Environmental Legislators . (2025). Confronting forever chemicals: States continue to lead the way in 2025. https://www.ncelenviro.org/articles/confronting‐forever‐chemicals‐states‐continue‐to‐lead‐the‐way‐in‐2025/

[jeq270279-bib-0036] Niedzwiecki, A. , Kates, N. , Brown, S. , & McIvor, K. (2022). Quantifying use in a community garden program with extensive resource provision to gardeners. Urban Agriculture & Regional Food Systems, 7(1), e20032. 10.1002/uar2.20032

[jeq270279-bib-0038] Pepper, I. , Kelley, C. , & Brusseau, M. (2023). Is PFAS from land applied municipal biosolids a significant source of human exposure via groundwater? Science of the Total Environment, 864, 161154. 10.1016/j.scitotenv.2022.161154 36572291

[jeq270279-bib-0039] Pepper, I. L. , Prasek, S. M. , Chorover, J. D. , Prevatt, F. J. , Barrientes, D. M. , Kester, G. , & Brusseau, M. L. (2026). National collaborative study on the incidence and mobility of PFAS following land application of biosolids. Science of the Total Environment, 1012, 181197. 10.1016/j.scitotenv.2025.181197 41421196

[jeq270279-bib-0040] Peter, L. G. , & Lee, L. S. (2025). Sources and pathways of PFAS occurrence in water sources: Relative contribution of land‐applied biosolids in an agricultural dominated watershed. Environmental Science & Technology, 59, 1344–1353. 10.1021/acs.est.4c09490 PMC11874022 39779456

[jeq270279-bib-0041] Sassi, M. (2024). Quality assurance project plan: Assessment of PFAS levels in biosolids in WA State (Ecology Publication 24‐07‐040). Department of Ecology, State of Washington. https://apps.ecology.wa.gov/publications/documents/2407040.pdf

[jeq270279-bib-0042] Sassi, M. , Kijowski, E. , & Corrman, A. (2025). Assessment of PFAS levels in Washington State biosolids (Publication 25‐07‐057). Department of Ecology, State of Washington. https://apps.ecology.wa.gov/publications/documents/2507057.pdf

[jeq270279-bib-0043] Shan, G. , Wang, Z. , Zhou, L. , Du, P. , Luo, X. , Wu, Q. , & Zhu, L. (2016). Impacts of daily intakes on the isomeric profiles of perfluoroalkyl substances (PFASs) in human serum. Environment International, 89–90, 62–70. 10.1016/j.envint.2016.01.002 26826363

[jeq270279-bib-0044] Simones, T. L. , Evans, C. , Goossen, C. P. , Kersbergen, R. , Mallory, E. B. , Genualdi, S. , Young, W. , & Smith, A. E. (2024). Uptake of per‐ and polyfluoroalkyl substances in mixed forages on biosolid‐amended farm fields. Journal of Agricultural and Food Chemistry, 72, 23108–23117. 10.1021/acs.jafc.4c02078 PMC11505395 39378197

[jeq270279-bib-0045] Sivaram, A. K. , Lee, E. , Curnow, A. , Surapaneni, A. , Kannan, K. , & Megharaj, M. (2023). Uptake, accumulation, and toxicity of per‐and polyfluoroalkyl substances in *Allium cepa* grown in soils amended with biosolids. Environmental Challenges, 10, 100670.

[jeq270279-bib-0046] Susmann, H. P. , Schaider, L. A. , Rodgers, K. M. , & Rudel, R. A. (2019). Dietary habits related to food packaging and population exposure to PFASs. Environmental Health Perspectives, 127(10), 107003. 10.1289/EHP4092 PMC6867167 31596611

[jeq270279-bib-0047] Tan, H. , Tang, S. , Yang, L. , Li, J. , Deng, Y. , Shen, H. , Dai, Q. , Gao, Y. , Wu, P. , Zhu, L. , & Cai, Z. (2024). Global quantification of emerging and legacy per‐ and polyfluoroalkyl substances in indoor dust: Levels, profiles and human exposure. Science of the Total Environment, 927, Article 172132. 10.1016/j.scitotenv.2024.172132 38569952

[jeq270279-bib-0048] Tittlemier, S. A. , Pepper, K. , & Edwards, L. (2006). Concentrations of perfluorooctanesulfonamides in Canadian total diet study composite food samples collected between 1992 and 2004. Journal of Agricultural and Food Chemistry, 54, 8385–8389. 10.1021/jf061713p 17032055

[jeq270279-bib-0049] Una, T. , Hernandez, J. , Beebe, A. , & Brown, S. (2022). How does your garden grow? Impact of residuals‐based amendments on urban soil health, vegetable yield and nutritional density. Urban Forestry & Urban Greening, 77, Article 127742. 10.1016/j.ufug.2022.127742

[jeq270279-bib-0050] US EPA . (2024). Method 1633, Revision A: Analysis of per‐ and polyfluoroalkyl substances (PFAS) in aqueous, soil, biosolids, and tissue samples by LC‐MS/MS . https://www.epa.gov/system/files/documents/2024‐12/method‐1633a‐december‐5‐2024‐508‐compliant.pdf

[jeq270279-bib-0051] US EPA . (2025). Draft sewage sludge risk assessment for perfluorooctanoic acid (PFOA) CASRN 335‐67‐1and perfluorooctane sulfonic acid (PFOS) *CASRN 1763‐23‐1* (EPA‐HQ‐OW‐2024‐0504; FRL 12451‐01‐OW). https://www.federalregister.gov/documents/2025/01/15/2025‐00734/draft‐sewage‐sludge‐risk‐assessment‐for‐perfluorooctanoic‐acid‐pfoa‐and‐perfluorooctane‐sulfonic

[jeq270279-bib-0052] USDA . (2017). Census of agriculture county profile: Douglas County, Washington . USDA. https://www.nass.usda.gov/Publications/AgCensus/2017/Online_Resources/County_Profiles/Washington/cp53017.pdf

[jeq270279-bib-0053] Venkatesan, A. K. , & Halden, R. U. (2013). National inventory of perfluoroalkyl sub‐stances in archived US biosolids from the 2001 EPA National Sewage Sludge Survey. Journal of Hazardous Materials, 252, 413–418. 10.1016/j.jhazmat.2013.03.016 PMC3776589 23562984

[jeq270279-bib-0054] Washington, J. W. , Yoo, H. , Ellington, J. J. , Jenkins, T. M. , & Libelo, E. L. (2010). Concentrations, distribution, and persistence of perfluoroalkylates in sludge‐applied soils near Decatur, Alabama, USA. Environmental Science & Technology, 44, 8390–8396. 10.1021/es1003846 20949951

[jeq270279-bib-0055] Wen, B. , Li, L. , Zhang, H. , Ma, Y. , Shan, X. , & Zhang, S. (2014). Field study on the uptake and translocation of perfluoroalkyl acids (PFAAs) by wheat (*Triticum aestivum* L.) grown in biosolids‐amended soils. Environmental Pollution, 184, 547–554. 10.1016/j.envpol.2013.09.040 24184376

[jeq270279-bib-0056] Whitehead, H. D. , Venier, M. , Wu, Y. , Eastman, E. , Urbanik, S. , Diamond, M. L. , Shalin, A. , Schwartz‐Narbonne, H. , Bruton, T. A. , Blum, A. , Wang, Z. , Green, M. , Tighe, M. , Wilkinson, J. T. , McGuinness, S. , & Peaslee, G. F. (2021). Fluorinated compounds in North American cosmetics. Environmental Science & Technology Letters, 8(7), 538–544.

[jeq270279-bib-0057] Yang, H. , Zhao, Y. , Chai, N. , Ma, F. , Yu, J. , Xiao, K. , & Gu, Q. (2024). Bio‐accumulation and health risk assessments of per‐ and polyfluoroalkyl substances in wheat grains. Environmental Pollution, 356, 124351. 10.1016/j.envpol.2024.124351 38878812

[jeq270279-bib-0058] Yoo, H. , Washington, J. W. , Jenkins, T. M. , & Ellington, J. J. (2011). Quantitative determination of perfluorochemicals and fluorotelomer alcohols in plants from biosolid‐amended fields using LC/MS/MS and GC/MS. Environmental Science & Technology, 45(19), 7985–7990. 10.1021/es102972m 21247105

